# An Incidental Finding of Scalp Angiosarcoma: A Case Report

**DOI:** 10.7759/cureus.13610

**Published:** 2021-02-28

**Authors:** Akram F Alwarqi, Mohammed Abdurabu, Pramod Gopalakrishnan, Yahya Paksoy, Munir Abu Ageila

**Affiliations:** 1 Radiology, Hamad Medical Corporation, Doha, QAT; 2 Emergency Medicine, Hamad Medical Corporation, Doha, QAT; 3 Internal Medicine, Hamad Medical Corporation, Doha, QAT

**Keywords:** angiosarcoma, scalp hematoma, transitional bladder cancer, endothelial tumor.

## Abstract

Scalp angiosarcoma is a malignant tumor of the vascular endothelial cells. We present the case of an elderly male patient with a history of urinary bladder transitional cell cancer and trauma (falling on his head) who came to the emergency department with scalp swelling, which was found on brain imaging to infiltrate into the skull, reaching the dural matter. A biopsy was done, which showed angiosarcoma, which is rare for that area. Further studies are recommended to establish if there is a possible genetic association between both cancers (urinary bladder transitional cell cancer and scalp angiosarcoma) as both arise from endothelial cells.

## Introduction

Scalp angiosarcoma is a malignant tumor of the vascular endothelial cells. Angiosarcoma usually affects the face and scalp areas, mostly in elderly people. Overall, the head and neck sarcomas are less common, constituting less than 1% of all head and neck malignancies and fewer than 5% of all soft tumor sarcomas occurring in the head and neck, with only approximately 10% classified as angiosarcomas [1]. Angiosarcomas of the face and scalp are insidious, and their clinical presentation varies widely. Mostly, they start as an innocent-looking tumor. But they primarily run a very aggressive course and may metastasize through lymphatic or hematogenous routes. Surgical resection remains the cornerstone of therapy. Recently, the application of treatment options such as chemotherapy and radiation has been explored given the tumor is mostly diffuse and metastasized at the time of diagnosis [2]. In this report, we present a rare case of scalp angiosarcoma that was found incidentally in an elderly patient who had presented with an isolated head injury.

## Case presentation

Our patient was an 80-year-old male with a past medical history of diabetes mellitus (DM) and hypertension (HTN) controlled with medications. The patient also had a history of recurrent bladder transitional cell cancer: Ta-low grade. The patient presented to the ED with a complaint of headaches. On examination, swelling over his scalp was noted. As per the patient, the swelling first appeared two months back when he had fallen on his head after slipping over stair steps; however, he had not sought any medical advice for the same. On local examination, scalp tenderness, skin redness encompassing an area of about 7-8 cm, necrosis, and fluctuation of the left temporoparietal region were observed. Hence, a CT of the head was requested to rule out intracranial hemorrhage.

**Figure 1:**
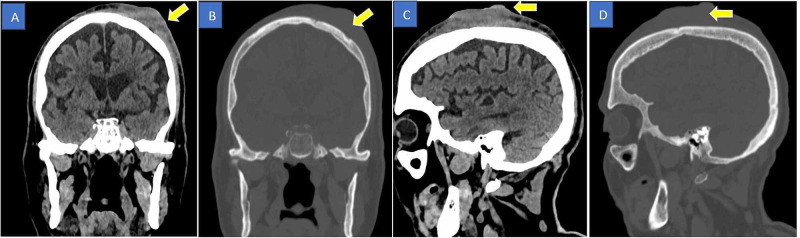
Plain CT head scan – coronal (A and B) and sagittal sections (C and D) The images show a left frontoparietal lesion with dermal and epidermal involvement causing bony erosion (yellow arrows) CT: computed tomography

**Figure 2:**
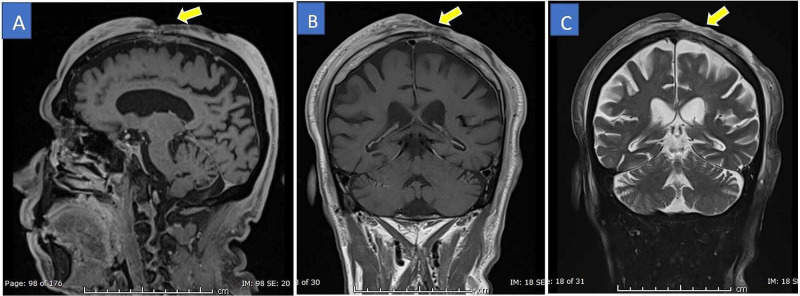
Brain MRI – sagittal T1-weighted image (A), coronal T1-weighted image (B), and coronal T2-weighted image (C) The images show an extensive bifrontal-parietal scalp lesion that is infiltrating the left frontal and parietal bone, passing through the calvarium, infiltrating the dura, and causing dural enhancement MRI: magnetic resonance imaging

The patient underwent a head CT scan, which showed a left frontoparietal lesion with dermal and epidermal involvement causing bony erosion (Figure 1). An MRI of the head was also performed (Figure 2). The patient underwent excision biopsy of suspicious scalp lesion with 5 mm margin with a split-thickness skin graft from his right thigh, which was performed by the plastic surgeon on 25/10/2020. Histopathology confirmed scalp angiosarcoma. Thereafter, the complete staging was done with an MRI brain and neck. The systemic staging was performed with CT of the chest, abdomen, and pelvis. There was no evidence of distant metastasis. After the completion of the studies, the case was referred to medical oncology for palliative chemotherapy and disease-specific follow-up for any evidence of disease progression.

## Discussion

Scalp angiosarcoma is an uncommon malignant tumor. In a majority of the cases, it is diagnosed incidentally when performing imaging studies for other diagnoses and diseases.

In this case, we encountered scalp angiosarcoma in an elderly man, who had a history of bladder cancer resected years ago, in the ED. The patient presented with an isolated head injury following a fall to rule out any bleeding. CT scan of the head showed no bleeding but revealed signs of scalp lesion. This is the most common presentation of scalp angiosarcoma, which is mostly found incidentally on a CT scan or MRI in an otherwise asymptomatic patient [3].

After finding this suspicious lesion, the patient was referred for more workup. MRI revealed an extensive bifrontal-parietal scalp lesion, which was passing through the calvarium, and infiltrating the dura and causing dural enhancement. This finding led to a biopsy, which showed evidence of scalp angiosarcoma. The biopsy is the primary diagnostic tool for this condition because CT scan and MRI have their limitations regarding diagnosing such conditions [4].

After the diagnosis, the patient was scheduled for radiotherapy in order to control the bleeding with a plan to do chemotherapy in the future. The benefit of surgical intervention was deemed minimal due to the wide diffusion to the scalp; there was a high risk of injury in addition to a high chance of damaging nearby structures. However, ironically, surgery is still the primary therapeutic choice for such patients, but most of them will not benefit from such an intervention [5].

This disease has a very poor prognosis, even with all the modalities of treatment on offer. Even though many studies and research on curative therapies are underway, the only treatment currently available is palliative, and others are mostly medications that are still in the trial stages [6].

The significance of our case lies in the fact that our patient was diagnosed with scalp angiosarcoma incidentally. He already had his bladder cancer resected years ago, which raises the question as to whether these two conditions are related. Both bladder cancer and scalp angiosarcoma arise from endothelial cells, and hence it is possible that there was a genetic element to his presentation or even a hereditary cause [7].

## Conclusions

We discussed the case of a patient who initially presented with head trauma, and underwent imaging that revealed infiltrating scalp angiosarcoma incidentally. As the patient had a history of urothelial cell cancer, and since both bladder cancer and angiosarcoma arise from endothelial cells, further studies are required to explore a possible genetic or hereditary association between these two cancers.
